# Adaptation and Validation of the Malay Version of the Index of Dental Anxiety and Fear (IDAF-4C^+^) for Malaysian Secondary School Children

**DOI:** 10.21315/mjms2018.25.3.11

**Published:** 2018-06-28

**Authors:** Izyan Hazwani Baharuddin, Wan Nor Arifin, Yee Cheng Kueh, Normastura Abd Rahman

**Affiliations:** 1 Unit of Biostatistics and Research Methodology, School of Medical Sciences, Universiti Sains Malaysia, 16150 Kubang Kerian, Kelantan, Malaysia; 2 Dental Public Health Unit, School of Dental Sciences, Universiti Sains Malaysia, 16150 Kubang Kerian, Kelantan, Malaysia; 3 Faculty of Dentistry, Universiti Teknologi MARA, Sungai Buloh Campus, Jalan Hospital, 47000 Sungai Buloh, Selangor, Malaysia

**Keywords:** dental anxiety, dental fear, validation studies, reliability, children

## Abstract

**Background:**

Many questionnaires have been developed to measure dental anxiety and fear. Among them is the Index of Dental Anxiety and Fear Scale (IDAF-4C^+^), which consists of a dental anxiety and fear module (IDAF-4C), a phobia module (IDAF-P) and a stimulus module (IDAF-S). The objective of this research was to report the adaptation and validation of the IDAF-4C^+^ for Malaysian secondary school children.

**Methods:**

This was a cross-sectional validation study. The original English version of the IDAF-4C^+^ was translated into Malay, back-translated, and then sent for content validation via an expert validation and face validation by the target student population. Three hundred and seventy questionnaires were then distributed among 16-year-old school children. Confirmatory factor analysis (CFA) was conducted for the IDAF-4C module using a bootstrapped maximum likelihood estimator. Spearman’s rank correlation was used to assess the relationship between the IDAF-S and IDAF-4C modules. Intraclass correlation (ICC) was used to determine the stability of the IDAF-S and IDAF-4C modules, while kappa values were used for the IDAF-P module.

**Results:**

The response rate was 86.5% for CFA and 76.9% for stability. CFA showed the existence of only one factor with a reliability estimate of 0.921, obtained via Raykov’s procedure. All items in the IDAF-S module were significantly correlated with the IDAF-4C module (*P* < 0.001). The IDAF-S and IDAF-4C modules were stable, as determined via a two-way mixed model with absolute agreement, a single measure and a Case 3 ICC (A, 1). The IDAF-P module showed satisfactory stability, as assessed via kappa values.

**Conclusion:**

The Malay version of the IDAF-4C^+^ is valid and reliable in measuring dental anxiety and fear among Malaysian secondary school children.

## Introduction

The Oral Health Division of the Malaysian Ministry of Health has been providing dental services in the form of three components, primary oral healthcare, specialist oral healthcare and community oral healthcare (1), with around 1,680 dental facilities and 3,127 dental units as of 2014 (2). Within the primary oral healthcare alone, services are provided to toddlers, antenatal mothers, the elderly and children in kindergarten, as well as school dental services and outpatient services (3). Having been exposed to many dental programs since childhood, Malaysians are expected to continue to utilise dental facilities when they grow up, without experiencing any fear or anxiety regarding such treatments. Surprisingly, the 2014 annual report by the Oral Health Division revealed that primary oral healthcare is only utilised by 25.2% of the population. Only 8.3% of adults, 8.0% of the elderly and 39.2% of antenatal mothers utilise primary oral healthcare, as compared to 98.4% of primary school children and 90.1% of secondary school children. In Negeri Sembilan alone, there were only 91,321 adults and 13,007 elderly people who utilised the service (2), though the total population of this state is 1.04 million (4).

A review of oral healthcare in Malaysia reported 47% of the adults had not visited the dentist for more than two years and 6% had not visited at all (5). It was also found that fear contributes to about 8.6% of the delays in the utilisation of dental facilities in Malaysia (6), which likely affects quality of life (7) and has other psychosocial consequences (8, 9). Many Malaysians may develop oral health problems, such as dental caries and periodontal disease (10). In terms of dental health, a study on the relationship between dental anxiety and dental caries in antenatal mothers revealed that participants with higher levels of dental anxiety displayed poorer oral health status than those with moderate and lower levels of dental anxiety (11). Similar findings were demonstrated by Klingberg (12) in his study of dental fear and fear management problems in children. In addition, dental fear has also led to a significant problem in terms of patient management because such patients are prone to cancel their dental appointments (13).

Due to the implications of dental fear reported in earlier research, it is important for us to determine who has dental anxiety and fear, as well as the nature and extent of their fear, so that further actions can be taken to overcome such situations. For that purpose, many scales and questionnaires have been developed to measure dental anxiety and fear. Among the most widely used are Corah’s Dental Anxiety Scale (DAS), Kleinknecht’s Dental Fear Survey (DFS), Gale’s Ranking Questionnaire (RQ) and Stouthard’s Dental Anxiety Inventory (DAI). While these questionnaires provide a basis for an appreciably expanded understanding of dental anxiety and fear, an appraisal review has shown some drawbacks with these early questionnaires regarding the data collected, reliability, validity, normative scores, correlation between questionnaires, ambiguity, absence of manuals and whether the questionnaire addresses the three elements of dental anxiety and fear that have been identified on theoretical grounds. The scales either have poor construct validity or are unrelated to dental fear. They only measure the emotion of fear, excluding its physiological, behavioural and cognitive components; the fear stimuli rather than the fear itself; or take so long as to make them impractical (14). Thus, the Index of Dental Anxiety and Fear Scale (IDAF-4C^+^) was developed in 2010 (15).

There is a lack of instruments that are culturally adapted to measuring dental anxiety and fear among the Malay-speaking population in Malaysia. Hence, the cultural adaptation and validation of these instruments is necessary. The aim of this paper was to report the adaptation and validation of the IDAF-4C^+^ for Malaysian secondary school children.

## Materials and Methods

### Study Design and Participants

This was a cross-sectional study. Five schools in the Rembau district were selected via stratified cluster random sampling and stratified by school category, following the list obtained from the Ministry of Education. One school was excluded because it had already been involved during the face validation phase. The class was set as the primary sampling unit, with the number selected being proportionate to the school size. The cluster size was estimated to be 26, which was obtained by dividing the total number of Form 4 students in the Rembau district (1,318) by the total number of Form 4 classes (52). Only Form 4 students were included in this study, while the illiterate and non-Malaysian were excluded. Data were collected from August until November 2014.

The sample size was determined based on a 20% non-response rate and a cluster effect of 1.5. Using an N:q ratio of 10:1, where q is the freely estimated parameter (16), a minimum of 360 samples (14 clusters) were required. To measure the stability of the questionnaire, 143 samples (six clusters) were needed, as calculated via a sample size calculator (17) with ρ_0_ set at 0.5 and ρ set at 0.7.

### English Version of the IDAF-4C^+^

Armfield (15) began the development of the original English questionnaire in 2008 in Australia. It consists of 23 questions categorised into three independent modules, namely the IDAF-4C (dental anxiety and fear module), IDAF-P (phobia module) and IDAF-S (stimulus module). The IDAF-4C is the core module, and the “+” sign indicates the presence of the other two modules.

The IDAF-4C was designed to measure dental anxiety and fear. It contains eight questions distributed into the following four domains that measure dental anxiety and fear: emotional, behavioural, physiological and cognitive. Each domain is represented by two questions. The responses are in the form of a 5-point Likert scale ranging from “disagree” to “strongly agree.” The overall scale score for this module can be calculated by averaging the total score (range: 1–5). The appropriate cut-off point was suggested to be between 2.5 to 3.5, depending on the researcher’s own judgement (18).

The IDAF-P contains five items and uses the DSM-5 diagnostic criteria (19) to enable a non-clinical diagnosis of a specific phobia along with a differential diagnosis for panic disorders and social phobias when used in conjunction with the IDAF-4C module. The questions in this module ask whether certain statements applied to the respondent or not. The answer is reported in a binary form: “Yes” or “No.” The determination of a potential dental phobia requires an indication of “marked fear” on the IDAF-4C module.

IDAF-S contains 10 items covering a range of stimuli most commonly reported to cause anxiety in the dental setting. It requires the respondent to record the extent to which he or she is anxious regarding a variety of dental stimuli on a response scale ranging from “not at all” to “very much.” All items are analysed individually. Therefore, the calculation of an overall score is not required.

### Translation, Back-Translation and Adaptation

The method for the translation and adaptation process was adapted from the World Health Organization for the Management of Substance Abuse Questionnaire (20). The questionnaire was forward-translated from English into Malay by the researcher herself, who had two years of experience in the translation field. The translated version was then emailed to two panels, each of which had medical and dental expertise. The expert panels then reviewed the translated Malay version and provided the necessary comments. At this stage, the communication was performed through email. Five corrections were made based on the comments. The corrected Malay version was then translated back into English by an independent language expert who had no knowledge of the original English version. A meeting was then conducted to discuss and finalise the questionnaire among all the personnel involved, including (a) the forward translator, (b) the medical and dental experts and (c) the backward translator. The expert panels also reviewed the content validity.

Following the guidelines by the World Health Organization for the Management of Substance Abuse Questionnaire (20), the questionnaire was pre-tested among 10 students from one of the schools to determine its face validity. These students were selected via convenience sampling. Because a few modifications were made based on the comments provided by the respondents, the questionnaire was then sent to be reviewed by another language expert, who was a teacher. At this stage, the parties communicated through email and phone. The questionnaire was then distributed for another face validity test using different students from the same school. The questionnaire received a good review and no further corrections were necessary.

### Data Collection

School principals and counsellors were given a briefing regarding the questionnaire and consent sheet so that they could provide proper instructions to the selected students. Based on these principals’ and counsellors’ advice, classes that did not fulfil the inclusion and exclusion criteria were excluded from the research. Because a self-administered questionnaire was used, the questionnaire was given to the school counsellor for distribution. Participation in this research was voluntary and parental permission was required. The questionnaire was to be completed by the students. Because implied consent was used, those who agreed to participate only needed to return the answered questionnaire after getting their parents’ permission and were allowed to keep the research information sheet for their records. The answered questionnaires were collected from the school counsellor after 10 working days. To measure the stability of the questionnaire, the test-retest was conducted two weeks after the collection of the first questionnaire. The same method and questionnaire were used. Students were identified via the identification number provided during the first data collection.

### Statistical Analysis

The data management and statistical analysis were performed using IBM SPSS Version 22 and SPSS Amos Version 20 (Amos).

Confirmatory factor analysis (CFA) was performed for the IDAF-4C module based on the results obtained from previous studies (15, 21). This test is superior to exploratory factor analysis (EFA) in terms of modelling flexibility and its ability to examine every potential source of invariance in the factor solution, including latent means and indicator intercepts (22). Univariate, bivariate and multivariate normalities, as determined via chi-square versus a Mahalanobis distance plot, were checked prior to the CFA analysis. All eigenvalues were positive definite, with a squared multiple correlation of < 0.90 and a variance inflation factor (VIF) of < 10. The scaling of the latent variable was performed by constraining the factor loading to 1.0 (22). Only overidentified models were evaluated. Modifications to the models were made based on standardised residuals, modification indices (MIs) and factor loadings > 0.5. Correlated errors were added based on the MIs, after considering the theory involved and the wording used. The number of bootstrapped samples was set to 250 (23). The fitness of the model was determining using relevant model fit indices. The construct reliability of the final CFA model was determined via Raykov’s procedure using a bootstrapping technique (24). Spearman’s rank correlation analysis was conducted between the mean total score of the IDAF-4C module and each individual item in the IDAF-S module to assess the strength and direction of the relationship between dental fear stimuli and the fear itself. For the IDAF-4C and IDAF-S modules, the test-retest reliability was determined using an intraclass correlation (ICC) two-way mixed model, absolute agreement, single measure, Case 3 ICC (A, 1). Kappa values were used for the IDAF-P module.

### Ethics

This study was approved by the Human Research Ethics Committee, Universiti Sains Malaysia (JEPeM code: USM/JEPeM/14070254). Permission to collect data involving school children was obtained from the Ministry of Education (KP[BPPDP]603/5/ JLD.10). Meanwhile, permission to translate this questionnaire was obtained from the original developer. This research was conducted in full accordance with the World Medical Association Declaration of Helsinki.

There was a total of 320 respondents who agreed to participate, as implied from the return of the answered questionnaires. The questionnaire was completed by the students anonymously, and information obtained was used only for the purposes of this research.

## Results

### CFA

A total of 370 self-administered questionnaires were distributed to students from the selected clusters. Only 320 answered questionnaires were returned, yielding a response rate of 86.5%. The demographic characteristics of 320 participants are summarised in Table 1.

**Figure 1:**
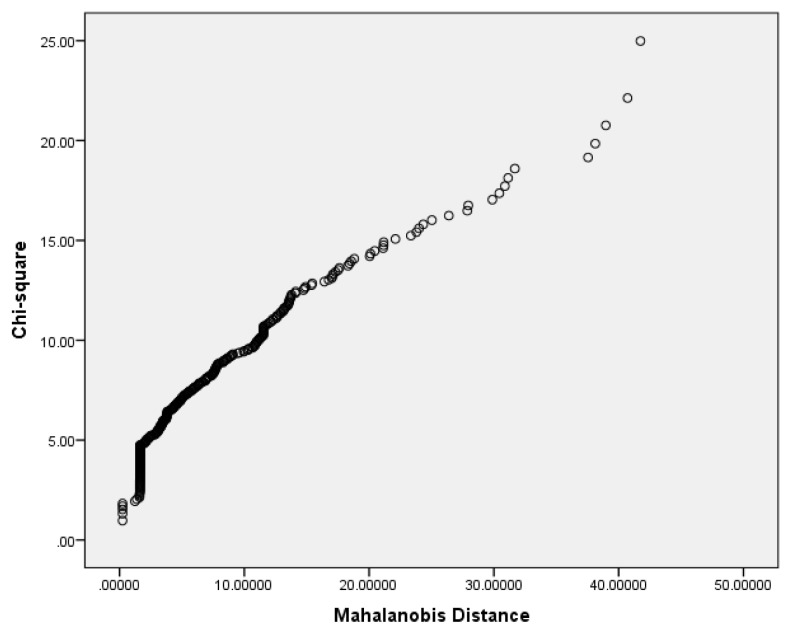
Chi-square versus Mahalanobis Distance plot

**Figure 2:**
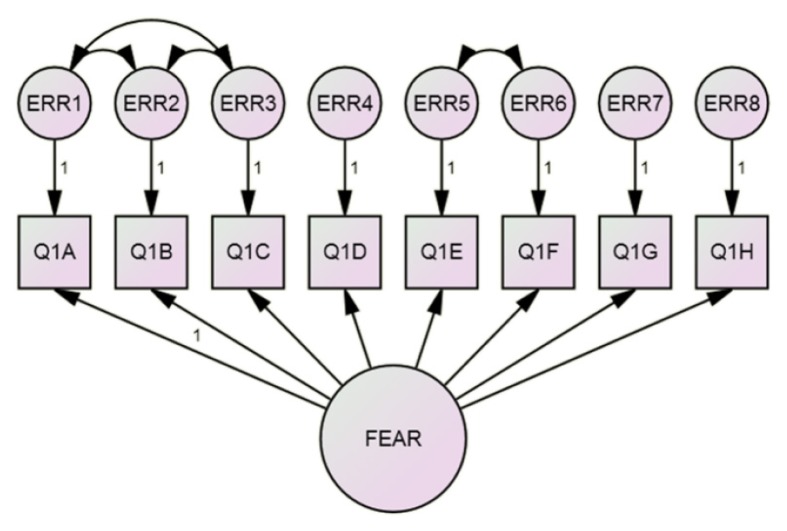
CFA for the IDAF-4C model

The assumption of multivariate normality was violated as shown by the chi-square versus Mahalanobis distance plot (Figure 1). The CFA for the IDAF-4C module was first conducted based on the four-factor model, as proposed by the developer (15). However, the solution for this model was not acceptable due to a negative variance. An analysis was then performed based on the one-factor model (21). Q1A was used as a marker indicator, and the factor loading was fixed at 1.0. This model was appropriate after some modifications (Figure 2). There were no standardised residuals of more than 1.96 and no covariance between errors. The bootstrap procedure for this model was successful in that the Bollen-Stine *P*-value showed a non-significant result (*P* = 0.251). The fit indices for this model are summarised in Table 2.

**Figure 3:**
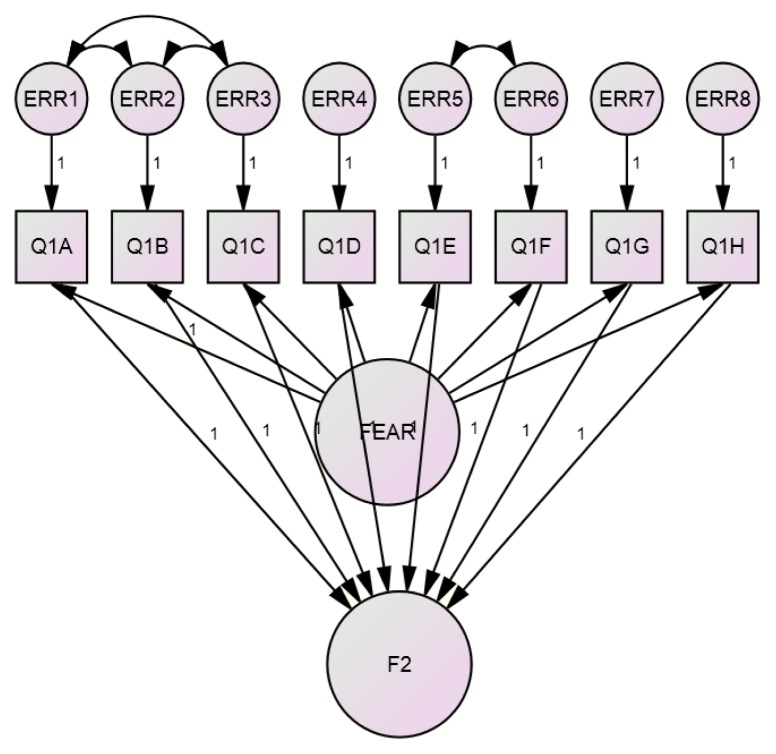
Realibility as determined via Raykov’s procedure

The construct reliability for the IDAF-4C, as determined via Raykov’s procedure (Figure 3), was 0.921 (90% CI = 0.904, 0.936; *P* = 0.006), indicating a good reliability.

### Correlation between the IDAF-4C and IDAF-S Modules

Spearman’s rank correlation yielded *r* values ranging from 0.278 to 0.566. Item Q3F (*Not knowing what the dentist is going to do*) had the highest correlation with the IDAF-4C module, while item Q3G (*The cost of dental treatment*) had the lowest correlation with the IDAF-4C module. All correlations were significant at the *P* < 0.001 level. The results are shown in Table 3.

### Stability of the Questionnaire

The IDAF-4C and IDAF-S modules displayed an acceptable level of agreement, ranging from fair to good as assessed via ICC. Meanwhile, for the IDAF-P module, two items displayed fair agreement, as assessed via kappa values. The results are summarised in Table 4.

## Discussion

The Malay version of the IDAF-4C^+^ produced valid and reliable measures of dental anxiety among secondary school children, as proven by the results of the EFA and Cronbach’s alpha values (21) and later confirmed by the findings of this study.

In the CFA, one factor, namely “fear,” was used, and this decision was supported by the high factor loading of each item in the EFA (21), as well as findings regarding the Spanish version (25). Only overidentified models were evaluated since under identified models cannot be solved while just identified models will always have a perfect fit, thus a model fit application was not applicable (22). A Bollen-Stine bootstrap provided the corrected *P*-values for the chi-square statistic, which allowed us to assess an overall model fit as compared to the usual maximum likelihood–based *P*-value (16, 22, 26). The addition of correlate errors during this analysis was based on the theoretical and empirical rationale. Q1A, Q1B and Q1C shared similar words and concepts. Based on the literature search, no other studies have validated the IDAF-4C module to the CFA level. The validations of both the original (15) and Spanish versions (25) reached only the EFA level, while the French (27) and German versions (28) could not be confirmed because the literature was not in English. Since the original version was not confirmed via CFA, this could be a cause of the variation in the number of factors when the module was validated in other languages.

Spearman’s rank correlation was used due to the violation in bivariate normality. All stimulus items correlate significantly with the IDAF-4C module, indicating an association between the stimuli measured and dental fear. Among the 16-year-old Rembau population, not knowing what the dentist is going to do is far more frightening than other dental stimuli. This result differs from those of both the Spanish (25) and English versions (15), suggesting that the difference could be due to age or geographical factors.

The stability results could not be directly compared with those of the English and Spanish versions because different analyses were used. In this study, the decision to use a two-way mixed model with absolute agreement, a single measure, and Case 3 ICC (A, 1) for the IDAF-4C and IDAF-4S modules was made because we were interested in the absolute agreement of the ratings given on two separate occasion by the same raters (29–31). This test is superior to the classical way of measuring agreement via Pearson’s correlation, which tends to yield results that are higher than the true level of agreement (32).

In this study, we managed to achieve the minimum sample size required. Several limitations were noted during the study. First, most of the respondents were Malay, and the needed sampling proportion for urban areas was not achieved. These conditions may have affected the generalisability of the current study findings. Second, the questionnaire administration method itself may have been a source of bias. Because the researchers had no direct contact with the respondents involved and were not present when the questionnaires were distributed, a response bias was possible. Third, it was noted that, due to limited samples, we were unable to replicate the results in multiple samples to demonstrate the stability of the results. Thus, we would like to suggest this for future research.

## Conclusion

The IDAF-4C module consists of only one factor that measures fear. The Malay version of the IDAF-4C^+^ is a valid and reliable measure of dental anxiety and fear among Malaysian secondary school children.

## Figures and Tables

**Table 1:** Demographic characteristics of participants for confirmatory phase, CFA (*n* = 320)

Variable	*n* (%)
School category	
Urban	82 (25.6)
Rural	238 (74.4)
Gender	
Male	156 (48.8)
Female	164 (51.2)
Ethnicity	
Malay	297 (92.8)
Chinese	12 (3.8)
Indian	11 (3.4)

**Table 2:** Fit indices for the IDAF-4C model

Fit indices	IDAF-4C
TLI / CFI	0.976 / 0.986
RMSEA (95% CI) / Clfit *p*-value	0.061 (0.033,0.088) / 0.24
SRMR	0.0278
χ^2^ (DF) / *P*-value	34.688 (16) / 0.004
CMIN / DF	2.168
Bollen-Stine *P*-value	0.251

**Table 3:** Correlation between the IDAF-4C and each item in the IDAF-S module (*n* = 320)

Item in IDAF-S Module	Spearman’s rank correlation,* r*
Q3A	0.386
Q3B	0.437
Q3C	0.397
Q3D	0.407
Q3E	0.547
Q3F	0.561
Q3G	0.278
Q3H	0.496
Q3I	0.380
Q3J	0.393

**Table 4:** Stability as determined via ICC and kappa values

Module	Question	Cronbach’s alpha	ICC* (95% CI)	Kappa, κ	Strength of agreement
IDAF-4C	Q1A-Q1H	0.807	0.675 (0.565, 0.762)	-	Good
IDAF-P	Q2A	-	-	0.499	Moderate
	Q2B	-	-	0.445	Moderate
	Q2C	-	-	0.388	Fair
	Q2D	-	-	0.294	Fair
	Q2E	-	-	0.577	Moderate
IDAF-S	Q3A	-	0.525 (0.383, 0.643)	-	Fair
	Q3B	-	0.579 (0.446, 0.686)	-	Fair
	Q3C	-	0.552 (0.415, 0.665)	-	Fair
	Q3D	-	0.438 (0.282, 0.572)	-	Fair
	Q3E	-	0.576 (0.444, 0.684)	-	Fair
	Q3F	-	0.712 (0.612, 0.791)	-	Good
	Q3G	-	0.542 (0.403, 0.657)	-	Fair
	Q3H	-	0.670 (0.553, 0.760)	-	Good
	Q3I	-	0.664 (0.551, 0.753)	-	Good
	Q3J	-	0.633 (0.491, 0.738)	-	Good
